# Low-dose cone-beam CT (LD-CBCT) reconstruction for image-guided radiation therapy (IGRT) by three-dimensional dual-dictionary learning

**DOI:** 10.1186/s13014-020-01630-3

**Published:** 2020-08-12

**Authors:** Ying Song, Weikang Zhang, Hong Zhang, Qiang Wang, Qing Xiao, Zhibing Li, Xing Wei, Jialu Lai, Xuetao Wang, Wan Li, Quan Zhong, Pan Gong, Renming Zhong, Jun Zhao

**Affiliations:** 1 Department of Radiotherapy, Cancer Center, West China Hospital, Sichuan University, No.37 Guo Xue Alley, Chengdu, 610065 P. R. China; 2 The School of Biomedical Engineering, Shanghai Jiao Tong University, No. 800, Dongchuan Road, Minhang District, Shanghai, 610065 P. R. China

**Keywords:** Low dose, CBCT reconstruction, Radiation therapy, Dictionary learning

## Abstract

**Background:**

To develop a low-dose cone beam CT (LD-CBCT) reconstruction method named simultaneous algebraic reconstruction technique and dual-dictionary learning (SART-DDL) joint algorithm for image guided radiation therapy (IGRT) and evaluate its imaging quality and clinical application ability.

**Methods:**

In this retrospective study, 62 CBCT image sets from February 2018 to July 2018 at west china hospital were randomly collected from 42 head and neck patients (mean [standard deviation] age, 49.7 [11.4] years, 12 females and 30 males). All image sets were retrospectively reconstructed by SART-DDL (resultant D-CBCT image sets) with 18% less clinical raw projections. Reconstruction quality was evaluated by quantitative parameters compared with SART and Total Variation minimization (SART-TV) joint reconstruction algorithm with paired *t* test. Five-grade subjective grading evaluations were done by two oncologists in a blind manner compared with clinically used Feldkamp-Davis-Kress algorithm CBCT images (resultant F-CBCT image sets) and the grading results were compared by paired Wilcoxon rank test. Registration results between D-CBCT and F-CBCT were compared. D-CBCT image geometry fidelity was tested.

**Results:**

The mean peak signal to noise ratio of D-CBCT was 1.7 dB higher than SART-TV reconstructions (*P* < .001, SART-DDL vs SART-TV, 36.36 ± 0.55 dB vs 34.68 ± 0.28 dB). All D-CBCT images were recognized as clinically acceptable without significant difference with F-CBCT in subjective grading (*P* > .05). In clinical registration, the maximum translational and rotational difference was 1.8 mm and 1.7 degree respectively. The horizontal, vertical and sagittal geometry fidelity of D-CBCT were acceptable.

**Conclusions:**

The image quality, geometry fidelity and clinical application ability of D-CBCT are comparable to that of the F-CBCT for head-and-neck patients with 18% less projections by SART-DDL.

## Background

On-board CBCT which provides volumetric information of a patient at treatment position is valuable for accurate patient setup before the treatment. Among the multiple verification systems, CBCT has become the gold standard for IGRT [1]. Solid researches have revealed that IGRT reduced the risk of late complications [2] and improved clinical outcomes [3].

CBCT imaging would be repeatedly applied to a patient during the IGRT treatment course for over 25 times in common. It raises a great concern that repeated CBCT scans deliver too much dose to the patient at 3 ∼ 5 cGy per scan and 90 ∼ 150 cGy if scanned daily for Varian on-board imager [4] and the isocenter doses ranged between 0.1 and 2.2 cGy per scan for the Elekta X-ray volumetric imager [5]. In addition, CBCT delivery dose from a Varian Trilogy accelerator was accurately investigated by Monte Carlo simulations and their results have shown that doses from a typical head and neck CBCT scan to eye, spinal cord, brain and bone could be up to 8 cGy, 6 cGy, 5 cGy and even 25 cGy, respectively for Varian on-board imager [6]. Previous study pointed out that daily CBCT scan for IGRT could increase the secondary cancer risk by 2% up to 4% [7]. Therefore, there is great significance to reduce the CBCT delivery dose while remaining the reconstruction quality.

Performing low-dose CBCT (LD-CBCT) scan is a common way to alleviate the excessive dose problem. However, degraded image quality would interfere with the therapeutic guidance correction of oncologists. Iterative reconstruction serves as a promising approach for LD-CBCT image improvement by incorporating prior image information [8, 9], regularized [10] and penalized weighted scheme [11, 12], dictionary learning method [13]. All the studies yield relative advantageous reconstructions with quantitative measurements, but no clinical practices were applied using the techniques, and no clinical efficacy and practical image quality were approved especially in IGRT.

Inspired by the existing works, it is a feasible approach to recover low quality structures of anatomical shape and texture by learning from numerous previous CBCT images. However, image characteristics might be different between the conventional Feldkamp-Davis-Kress algorithm CBCT (F-CBCT) and the LD-CBCT reconstructed from few-view projections, such as the different noise level and the different tissue contrast. In this study, we considered to employ the dual dictionary learning (DDL) strategy, which takes advantages of a paired atom that consists of anatomical structures from both F-CBCT and LD-CBCT images. We investigated the feasibility of the proposed few-view CBCT imaging method, the joint DDL and SART algorithm (SART-DDL), and further evaluated its clinical efficacy and image quality according to the clinical practice routine which consists of the registration with planning CT, geometry fidelity test and five-grade objective grading by experienced radiation oncologists.

## Methods

### Patients and CBCT simulation

In this retrospective study, a total of 62 CBCT image sets for 42 head-and-neck patients (12 females and 30 males, mean [SD] age, 46.6 [10.9] years for females, mean [SD] age, 50.9 [11.6] years for males) treated with IGRT from February 2018 to July 2018 at West China Hospital were randomly collected. All CBCT scans were performed by an Elekta Synergy XVI 4.7 imaging system (Synergy, Elekta, Crawley, UK). Gantry rotated 360° in CBCT scan protocol referring to previous publications [11, 14]. Around 390 projections views were evenly acquired over the whole scanning circle. The collimator cassette S20 was used and the corresponding field of view was 26 cm. F-CBCT images were directly reconstructed by the conventional Feldkamp-Davis-Kress algorithm with 512 × 512 × 132 spatial dimension. LD-CBCT projections of equal-angular 320 views were interpolated and extracted from the raw data and were reconstructed from the joint SART-DDL algorithm (the resultant D-CBCT image sets).

### Dual-dictionary learning theory

In the case of CBCT scanning for head and neck patient radiotherapy, empirically, anatomy structures are mostly similar among different patients. It implies that the general anatomical features of a newly scanned CBCT is highly possible to be obtained from the previous CBCT image database of the same scanning field. Based on the assumption, taking advantages of such prior information is a feasible way to restore LD-CBCT image features. In our study, the dictionary of prior features is employed to improve LD-CBCT using under-sampled-view data.

Dictionary learning (DL) method pursues a sparse combination of dictionary atoms to represent the target image. The atoms are supposed to involve the most common image features trained from prior images to ensure the representation sparsity [15]. The sparse representation means linearly combining a few prior features to reproduce the complete target images in high quality. However, it is improper to use high-quality features to directly fit in a low-quality CBCT image representation because of their different resolutions. A dual-dictionary is exactly comprised of two strictly paired dictionaries with different properties, such as two modalities or resolutions [16]. Two sets of the reconstructions of the same CBCT scan, referring as a full view reconstruction (high-quality) and an under-sampled view reconstruction (low-quality) are adopted to train the dual-dictionary. The consistency of structure features between the two image sets guarantees a stronger correlation of the sparse representation than DL method.

### Few view CBCT imaging

We proposed SART-DDL joint reconstruction algorithm for few-view LD-CBCT. SART and DDL are alternatively implemented. SART first takes use of the cone beam few-view X-projection data and yield a low-quality CBCT image. Afterwards, DDL transits high-quality features to the low-quality image.

A dual-dictionary is constructed by performing K-Singular-Value decomposition algorithm [17], using elements of cubic image patch pairs extracted from high-quality and low-quality CBCT image pairs. The dictionary is fixed after the construction.

In our SART-DDL reconstruction experiments, the dual-dictionary was constructed from five prior CBCT scans. Dictionary elements in shape of patches of size 8 × 8 × 8 pixels were adopted. Few-view CBCT projections were reweighted in order to mitigate typical cone artifact [18]. The flowchart of complete algorithm is shown in Additional Figure 1. Target few-view images were initially produced by using SART reconstruction with 18% less under-sampled panel X-projections. SART-DDL were performed twice to fully import high-quality features. We also adopted two additional times of ordinary DL process joint with SART reconstruction for further denoising. The single dictionary was constructed using only high-quality images of the five scans. More information about the mathematical description of the DL theory and SART-DDL algorithm are provided in the Additional File.

### Image quality evaluation

For objective evaluation, quantitative metrics peak signal-to-noise ratio (PSNR) and structural similarity (SSIM) were applied to our experiment results for numerical comparisons between SART-DDL and SART-TV [19], which was typical iterative reconstruction algorithm. The PSNR and SSIM are commonly used standard metrics for quantitative analysis in CBCT reconstruction previously [20, 21]. In our study, the reference images were F-CBCT sets, which are widely adopted in present clinical applications and regarded as the basic standard. The details and comparisons of different algorithms are listed in Additional File Table 1.

CBCT requires high geometry fidelity to guarantee the registration accuracy. To evaluate the geometry fidelity, three dimensional scale was tested using Catphan CTP 503 phantom (CATPhan® Phantom Laboratory, Salem, NY) according to the XVI R4.5 Elekta Customer Acceptance Tests. For transverse vertical and horizontal direction, the specification is 117 mm ±1.0 mm and for sagittal geometric, the specification is 110 mm ±1.0 mm. The tolerance is equivalent to ±4 pixels.

For subjective evaluation, the image quality of D-CBCT and F-CBCT was graded respectively according to the key points of the clinical requirements of our department using a four-grade scale by two radiation oncologists independently in a blind manner. The selected criteria revealed the main aspects of the image quality (see Table 1). Scores of two sets of each patient were compared by paired Wilcoxon rank test and the statistical significance level was set at *P* < .05 (IBM SPSS, version 25.0, New York, USA).

**Table 1:** Five-grade subjective evaluation criterions

Index	Criterion	Scores				
		Strongly Agree	Agree	Neutrality	Disagree	Strongly disagree
1	The pterygopalatine fossa is clearly visible	5	4	3	2	1
2	The rupture hole is clearly visible	5	4	3	2	1
3	The boundary of pharyngeal fossa is clearly visible	5	4	3	2	1

### Clinical registration comparison

The aim of setup CBCT scanning is to verify the target position. We performed F-CBCT to planning CT registration and D-CBCT to planning CT registration respectively for each patient in a blind study. Each independent registration was operated in the registration module of Eclipse (v13.5, Varian Medical Systems, USA) treatment planning system. In the registration process, rigid bone auto registration algorithm was adopted. Registration results were verified by two radiation oncologists according to their consensus agreements. If disagreements occurred, decision would be done after one or more iterations of review and editing according to their discussion. Two groups of setup errors according to the registration results were recorded in x (transverse), y (superior-inferior), and z (anterior-posterior) directions, namely the translational localization error x_t-F_, x_t-D_, y_t-F_, y_t-D_, z_t-F_, z_t-D_ and the rotational localization error x_r-F_, x_r-D_, y_r-F_, y_r-D_, z_r-F_ and z_r-D_. Two sets of setup errors of each patient were analyzed by two-sided paired *t* test. To further evaluate the practical impact of the registration difference, the case with the maximum registration difference was selected to show the dose deviations according to the registration results.

## Results

**Fig. 1:**
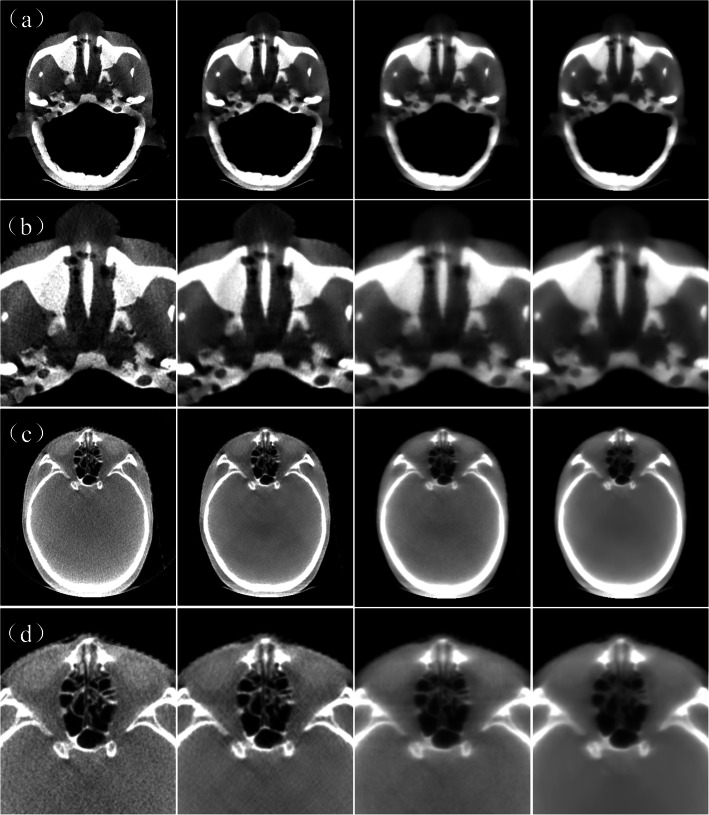
Comparison of representative slices of FDK, SART-DDL, SART and SART-TV. **a** shows typical slices at the same transverse location of an anthropomorphous head and neck phantom and **c** shows the reconstruction result of a typical patient. **b** and **d** shows the corresponding partial magnified parts of **a** and **c**, respectively. From left to right, the images are reconstructed by the FDK, SART-DDL, SART and SART-TV, respectively. **a** and (**b**) are displayed in the window of [− 220 HU, 720 HU]. **c** and **d** are displayed in the window of [− 450 HU, 550 HU]

Figure 1 shows the SART-DDL reconstruction results of an anthropomorphous head and neck phantom (see Fig. 1 (a) and (b)) and a typical patient (see Fig. 1 (c) and (d)). The PSNR of the SART-DDL phantom was 38.20 dB and the SSIM was 0.99. The corresponding PSNR and SSIM results of SART-TV reconstruction were 36.10 dB and 0.98, respectively. The PSNR of SART-DDL for the patient example was 2.7 dB higher than the SART-TV reconstructions.

Table 2 shows the quantitative comparison results of SART-DDL and SART-TV for 62 cases. For all CBCT image sets, the image qualities of D-CBCT were recognized as clinically acceptable by the two radiation oncologists. The median scores and the statistic comparison results are listed in Table 3. There was no significant difference between D-CBCT and F-CBCT.

**Table 2:** The subjective grading evaluation for LD-CBCT with 62 cases

	SART-DDL (dB)	SART-TV	*P*
PSNR	36.36 ± 0.55	34.68 ± 0.98	< .001
SSIM	0.99 ± 0.002	0.98 ± 0.001	< .001

**Table 3:** The subjective grading evaluation for LD-CBCT

Score		Criterion 1		Criterion 2		Criterion 3	
Image sets		F-CBCT	D-CBCT	F-CBCT	D-CBCT	F-CBCT	D-CBCT
Oncologist 1	median [IQR]	5[1]	4[1]	5[1]	5[1]	5[0]	5[1]
	*P* value	.08		>.99		.08	
Oncologist 2	median [IQR]	5[1]	4[2]	5[1]	4.5[2]	5[0]	4[1]
	*P* value	.06		.1		.1	

IQR is for interquartile range

**Fig. 2:**
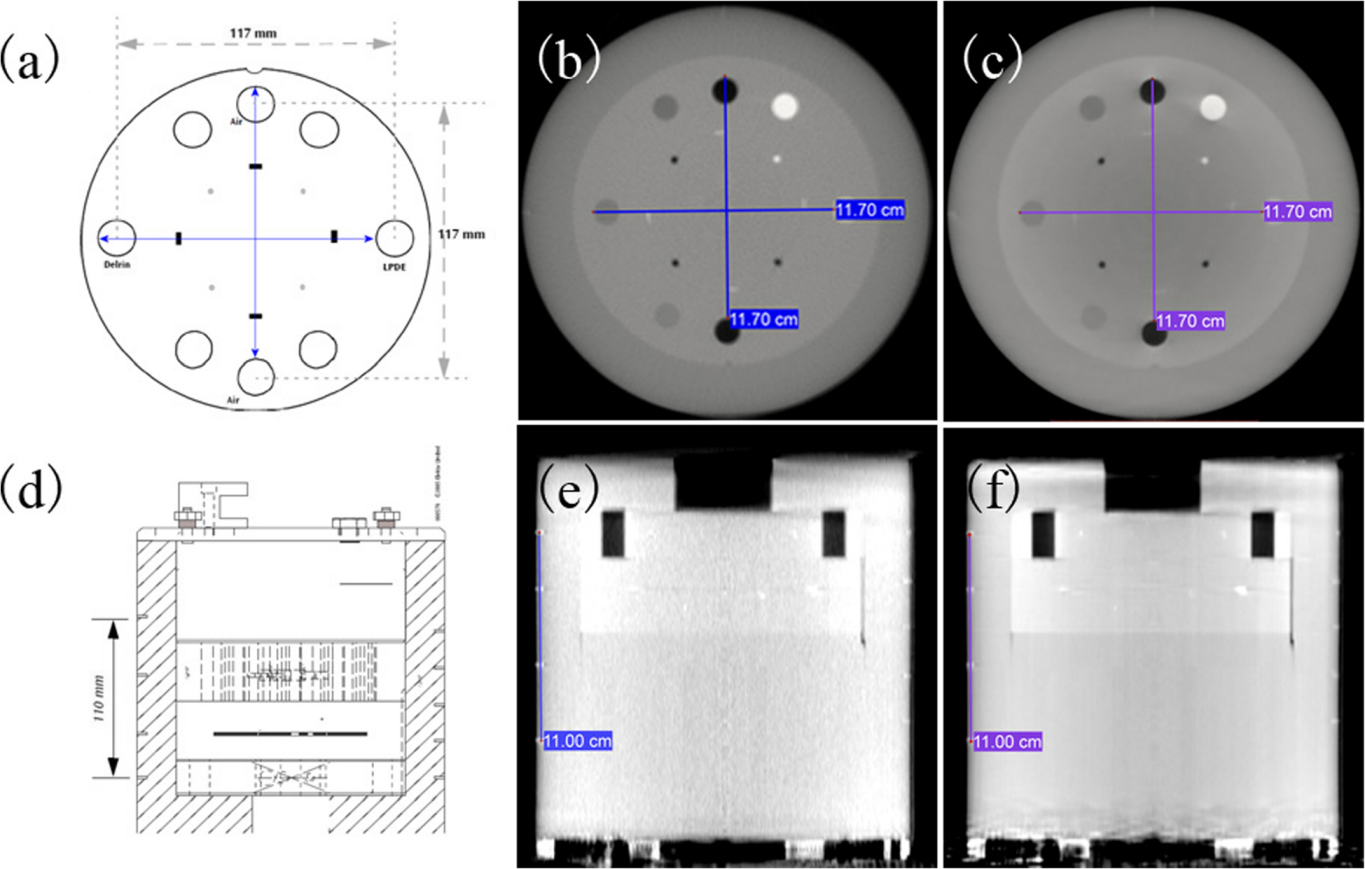
Geometry fidelity test of SART-DDL. The first row are the transverse vertical and horizontal scale specification diagram (**a**), transverse vertical and horizontal geometry test of F-CBCT (**b**) and D-CBCT (**c**). The second row are the sagittal geometric scale specification diagram (**d**), sagittal geometry test of F-CBCT (**e**) and D-CBCT (**f**)

Geometry fidelity test result is illustrated in Fig. 2. The length of the lines in Fig. 2 (a) was 117 mm, and the length for the lines in Fig. 2 (d) was 110 mm. Figure 2 shows that when the length was fixed, the start points and the end points of the lines were at the required locations according to the specification diagram. The results shows that the three-dimensional geometry fidelity of D-CBCT was in specification.

The statistical results of the registration experiments are exhibited in Table 4. The paired *t* test analysis of the registration setup error between the two groups showed statistical significance (*P*<.05), but the mean translational difference was limited to 0.30 mm and the mean rotational difference was 0.1°among three axes.

**Table 4:** The statistical results of the registration between F-CBCT and D-CBCT

	Translation (mm)			Rotation (°)		
	x_t-F_ - x_t-D_	y_t-F_- y_t-D_	z_t-F_ - z_t-D_	x_r-F_ - x_r-D_	y_r-F_ - y_r-D_	z_r-F_ - z_r-D_
Mean	0.10	0.20	−0.30	0.10	−0.1	−0.10
standard deviation	0.43	0.29	0.56	0.21	0.36	0.20
Absolute maximum	1.2	1.2	1.8	0.7	1.7	1.3
*P*	**.03**	**.001**	**.001**	**.001**	**.04**	.05

**Fig. 3:**
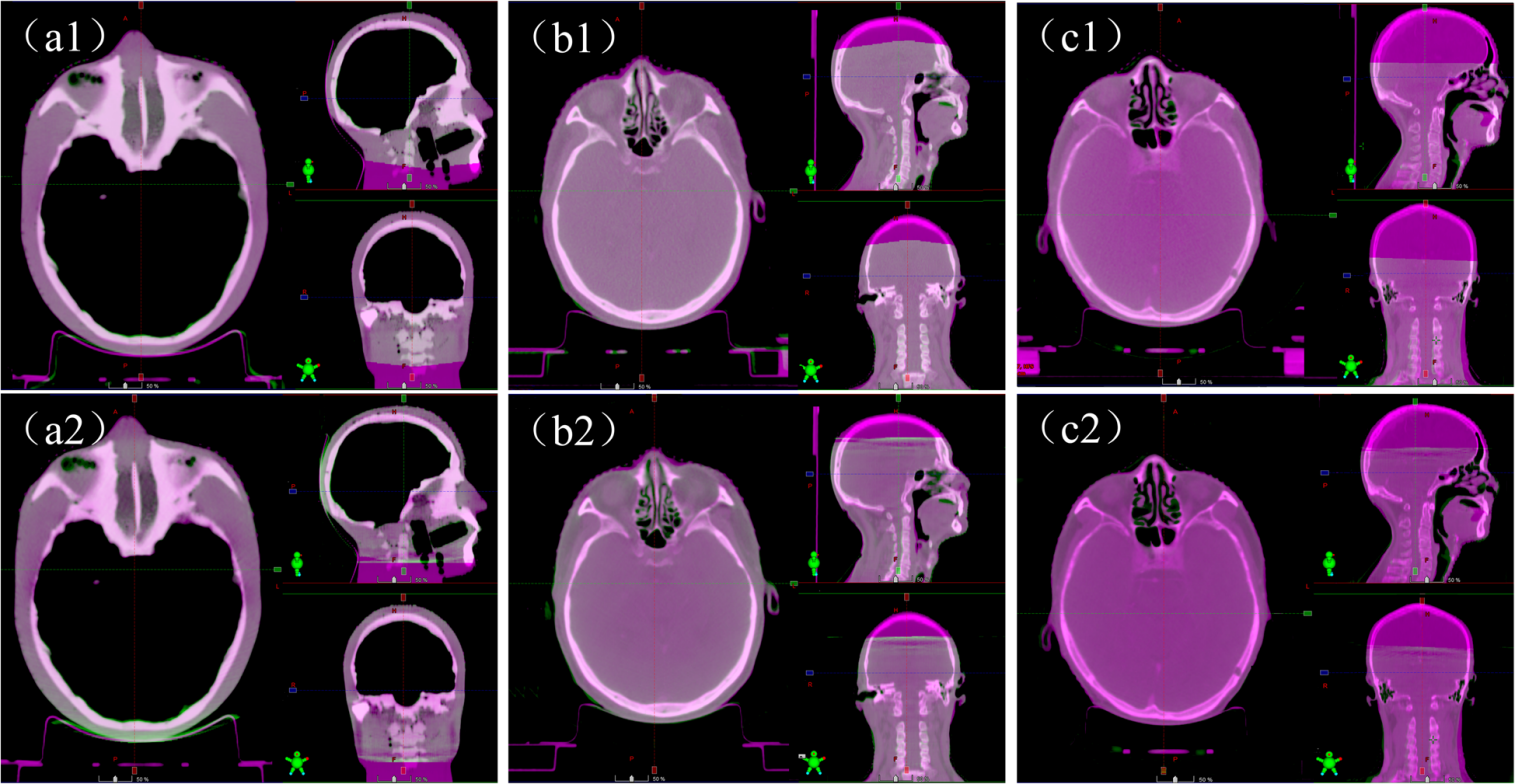
The illustration of three pairs of CBCT to planning CT registration. **a** shows F-CBCT to planning CT registrations (upper) and D-CBCT to planning CT registration (lower) of a head phantom. The translation differences were − 0.2 mm, − 0.1 mm and − 0.2 mm for x, y and z directions, respectively. The rotational differences were 0.1°, − 0.1°and − 0.1°for x, y and z directions, respectively. **b** shows F-CBCT to planning CT registrations (upper), and D-CBCT to planning CT registration of a typical case 1 (lower). The translation differences were − 0.2 mm, 0.2 mm and − 0.3 mm for x, y and z directions, respectively. The rotational differences were 0.2°, − 0.1°and − 0.0°for x, y and z directions, respectively. The registration result of case 1 was close to the average performance among 62 cases. **c** shows F-CBCT to planning CT registrations (upper) and D-CBCT to planning CT registration (lower) of a typical case 2. The translation differences were 0.4 mm, − 0.1 mm and − 1.8 mm for x, y and z directions, respectively. The rotational differences were − 0.1°, − 0.3°and − 0.2°for x, y and z directions, respectively. The registration difference of case 2 was the maximum among 62 cases

**Fig. 4:**
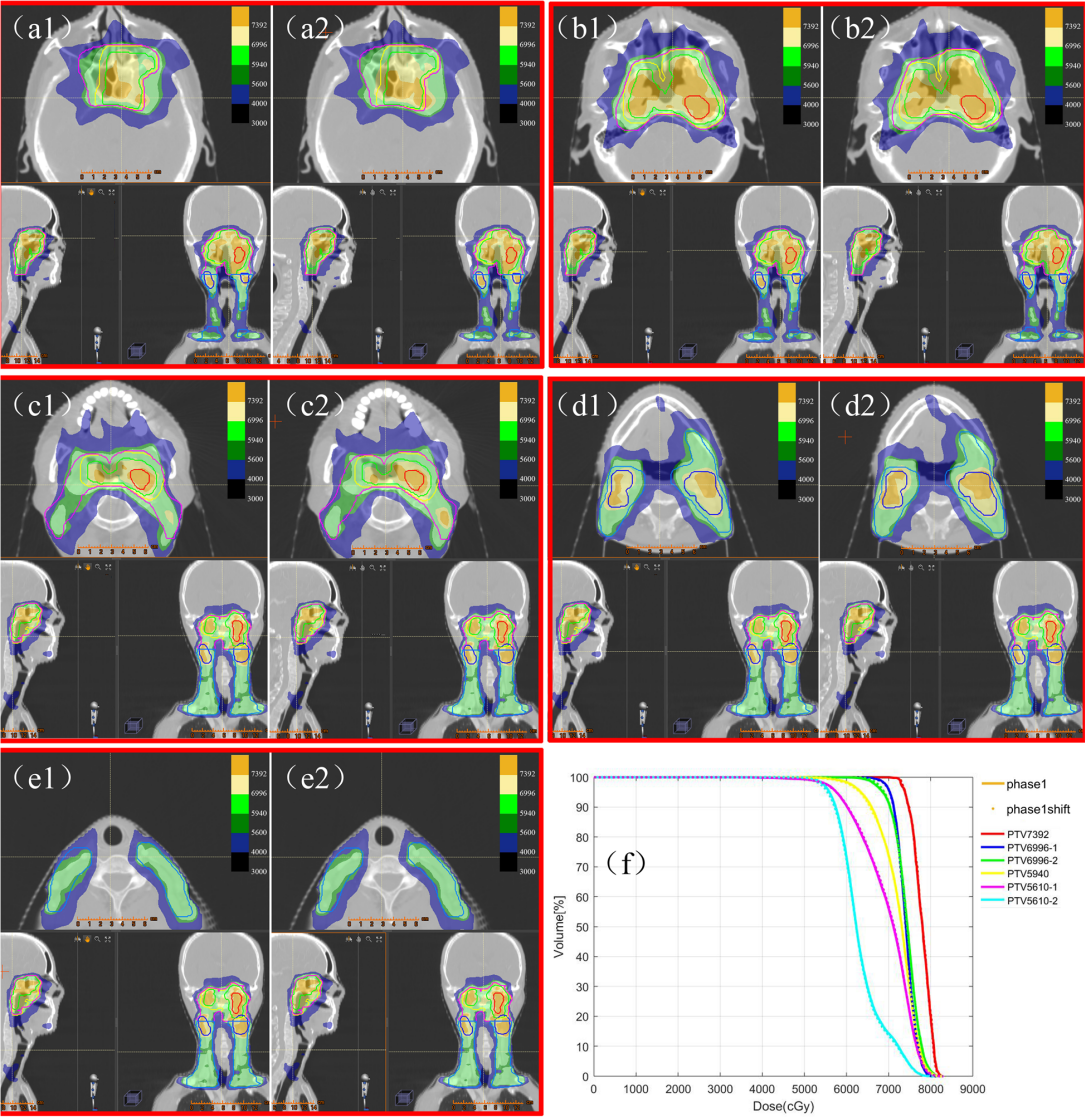
Dose comparison of the original plan and the shifted plan of case 2. (a1), (b1), (c1), (d1) and (e1) are multiple slices of dose distribution of the original plan. (a2), (b2), (c2), (d2) and (e2) are the dose distribution at the same location of the shifted plan according to the registration difference between F-CBCT and D-CBCT. (f) is the dose–volume histogram of the target volume from the original plan and the shifted plan

Three typical registration examples are illustrated in Fig. 3. The registration differences of the rigid phantom were limited to 0.2 mm and 0.1° (see Fig. 3 (a)). The registration result shown in Fig. 3 (b) was close to the mean level of the test cases. The registration difference illustrated in Fig. 3 (c) was the maximum among 62 cases. To evaluate the actual dose deviation caused from the registration difference, the shifted plan was done in Raystation treatment planning system (version 4.7.5, RaySearch Laboratories, Stockholm, Sweden) and was accepted by the responsible oncologist. The original and the shifted dose distribution according to the translational registration result of case 2 are illustrated in Fig. 4. The key dose parameters of the regions of interest and the dose difference maps are listed in Additional File Table 2 and Figure 2. Among all the key dose parameters for target volumes, the parameter that varied most was D99 of PTV5610–2, but the variation was also limited to 2% with the prescription of 74 Gy/33 fraction.

## Discussion

Our work implemented experiments on LD-CBCT IGRT clinical practice with the proposed SART-DDL. We used only five candidate head image sets which consists of a high quality CBCT and a strictly corresponded low quality CBCT. Five sets are empirically sufficient to construct a detailed dual-dictionary, because medical images consist of numerous repetitive features. Anatomical features in the dual-dictionary implicitly represent common CBCT head and neck image characteristics. In DDL reconstruction, image quality of the new D-CBCT set can be improved by replacing its low quality characteristics with the corresponding high quality structures through the correlation of the pair dual-dictionary atoms.

We compared our SART-DDL method with FDK and SART-TV. Inadequate projections undoubtedly degrade the reconstruction with blurred structures and streak artifacts. SART-DDL could effectively remove such noise and maintain anatomical structures. Compared with the non-prior-based traditional iterative reconstruction method SART-TV [19], DDL introduced additional high-quality anatomical information into D-CBCT images and improved reconstruction quality.

As for the state of art LD-CBCT reconstruction publications, penalty weighted regularized iterative reconstruction acquired 226 views in a head phantom scanning [11] . However, no quantitative measurements were evaluated and practical case studies in the conventional clinical routine were inadequate. Prior contour constrained few-view LD-CBCT reconstructions acquired 41, 50, and 62 views respectively to explore the imaging quality of brain scanning, but no quantitative measurements were mentioned and only one clinical case was tested [9]. Another algorithm imposed a single dictionary and did LD-CBCT reconstructions using 360 (PSNR = 35.98 dB), 180 (PSNR = 35.06 dB) and 120 (PSNR = 34.41 dB) projections for abdomen scanning imaging [13], but the data was theoretically simulated and inadequate for the statistical analysis. Clinical situations would be much complicated with various interferences. The effectiveness and robustness of the methods need further experiments in practice. In our study, raw projection data sets were adopted to take practical deviation factors into consideration, for example, scanning target movement, gantry mechanical vibrations, isocenter shifting, detector bias voltage drift, et al. On the basis of providing a qualified clinical protocol for IGRT, we determined to adopt the 18% reduced raw data in SART-DDL reconstruction with the qualified imaging quality. We also tried to use sparser projection views to explore the possibility of further radiation reduction, however, the reconstruction results were not clinically acceptable due to the details of anatomical structures cannot be discriminated clearly, such as the pharyngeal crypt, the eustachian tube orifice and the sphenoid sinus edge.

Besides undersampling the projection number, dose reduction can be achieved by lower mAs compared to a normal dose scan, which also causes image quality deterioration. In this study, we used a low mA (10 mA) and low mS (10 mS) scanning mode for all image data according to our clinical routine. However, in other studies, the higher mA and mS were used. For example, 33 mA/15 ms was used as the clinical protocol and 10 mA/10 ms as the low dose test protocol [9]. This comparison reveals that our clinical CBCT scanning baseline is in fact a low-dose protocol in the aspect of mAs and SART-DDL further reduced the scanning dose by sparse projections but not compromised the image quality. Another straightforward way to reduce imaging dose to patients is to employ flexible imaging protocols. For example, applying more kV-kV imaging would significantly reduce the imaging dose. However, the disadvantage of kV-kV imaging is obvious with only two dimensional information.

The SART-DDL reconstruction qualities were approved by quantitative measurements, the geometry fidelity test and subjective grading. No clinical difference was found. The noise level of D-CBCT was slightly higher than F-CBCT in some soft tissue region. Both F-CBCT and D-CBCT can effectively show the details of bony structures such as the skull, sphenoid sinus, maxillary sinus and ethmoid sinus well. The structure boundaries and bone walls were clear enough to be visually distinguished. There was no visual difference in terms of those bony structures among D-CBCT, F-CBCT and the planning CT. As for more details of relative small rigid structures, D-CBCT and F-CBCT can distinguish the nasal septum, the superior nasal concha, the sieve plates, the infraorbital ethmoid cell and the recessus sphenoethmoidalis. The boundaries of these structures on D-CBCT were relatively ambiguous, but it had no influence on clinical practices.

Registration results, which provides an important basis for clinical decision-making in error correction, were compared between F-CBCT and D-CBCT. In order to avoid the registration inconsistency from irrelevant factors such as the patient local deformation [22], the region of registration area was restricted to the area above the skull baseline. Maximum translation error was 1.8 mm and the maximum rotation error was 1.7° in the comparative analysis. The case with maximum registration difference was provided to reveal the SART-DDL practice impact by calculating the shifted dose. The dose distribution of the shifted plan changed little for target volumes. Therefore, it can be considered that the clinical results of registration using the two sets of images were consistent for head and neck patients.

It is worth noting that the good consistency of registration results in head and neck does not means it would be successful in other site (such as lung), because the registration in this study is based on bony structures. We will extend SART-DDL for thorax and abdomen patients and further explore the corresponding registration consistency in the future.

In consideration of the consumed time during the proposed SART-DDL reconstruction, we counted the time for each iteration in our experiments. The reconstruction was implemented using eight GTX-1080Ti graphic processing units (GPUs) and CUDA 7.5 platform. SART process was parallelized in terms of multiple X-rays. DDL was parallelized mainly in representation calculation (orthogonal matching pursuit algorithm). The details of the parallelization scheme can be found in the Additional File. The accumulative process time of SART (5 times) was 305 s, in the framework of 64 (Block) × 256 (Thread) × 8 (GPU) parallelization. The accumulative process time of DDL reconstruction (4 times) was 7072 s, in the framework of 128 (Block) × 64 (Thread) × 8 (GPU). The total CBCT reconstruction time was 7377 s (about 2 h). It should be noted there is still high potential to further accelerate the reconstruction. One scheme is to adopt advanced parallelization method to improve the calculation efficiency. For example, separable footprints in forward and backward projection would be much faster than Siddon’s projection calculation in our experiments [23]. Another scheme is to improve the computational ability, such as using Tesla P100 or K80. The recently developed technique “Cloud Computing” would maximize the computational ability without hardware limitation. Another relatively mature technique Field programmable gate array (FPGA) could also achieve a faster processing speed than GPU in DL process [24]. We are working on FPGA realization of SART-DDL and we believe there would be better acceleration solution in the future.

## Conclusions

In conclusion, the image quality and image registration application ability of D-CBCT is comparable to that of the F-CBCT for head and neck patients. SART-DDL LD-CBCT is a promising tool for clinical IGRT practice.

## Supplementary information

**Additional file 1.** Mathematical description of dual-dictionary learning theory (DDL).

## Data Availability

The datasets used and/or analysed during the current study are available from the corresponding author on reasonable request.

## Abbreviations

CBCT: cone beam CT
SART: simultaneous algebraic reconstruction technique
DDL: dual-dictionary learning
LD-CBCT: low-dose CBCT
TV: total variation
IGRT: image guided radiation therapy
D-CBCT: SART-DDL CBCT
F-CBCT: FDK CBCT
PSNR: peak signal-to-noise ratio
DL: dictionary learning
SSIM: structural similarity
GPU: graphic processing units
