# Supplementary material for: Low-dose cone-beam CT (LD-CBCT) reconstruction for image-guided radiation therapy (IGRT) by three-dimensional dual-dictionary learning
Source: Radiat Oncol. 2020 Aug 12;15:192. doi: 10.1186/s13014-020-01630-3 (PMC7425566; doi:10.1186/s13014-020-01630-3)
Supplement: Supplementary file 1 — Additional file 1. Mathematical description of dual-dictionary learning theory (DDL). [file 13014_2020_1630_MOESM1_ESM.docx]

**Additional File**

**Mathematical description of** **dual-dictionary learning theory (DDL)**

DDL can be described Mathematical as follows:

$\begin{matrix} x_{low}=D_{low}\alpha+\delta\\ x_{high}=D_{high}\alpha+\delta' \end{matrix}$ (1)

Where $x_{high}$ and $x_{low}$ are high-resolution and low-resolution CBCT images, respectively. $D_{high}$ and $D_{low}$ are the two paired dictionaries of high quality and low quality, respectively. $\alpha$ is the sparse representation of image $x$. $\delta$ and $\delta'$ are dictionary representation noise. According to strictly paired features in $D_{high}$ and $D_{low}$, it is reasonable to share the representation $\alpha$ in the dictionary learnings of both resolution levels. Transitivity of high image quality can be achieved by the shared representation and few-view CBCT reconstruction is able to be improved, with less artifact and more structural details.

**Mathematical description of SART-DDL**

Sparse representation $\alpha$ for property transitivity is calculated as follows:

(2)

where $x_{low}$ and $D_{low}$represent the low-quality CBCT image and the low-quality part in the dual dictionary; is a extraction function that divides the image into much smaller feature patches; $L$ is the upper limit of sparsity ($l_{0}$ norm) of the representation $\alpha$. Equation (2) can be solved by using orthogonal matching pursuit algorithm. A high-quality image $x_{high}$ can subsequently be obtained by applying high-quality dictionary part $D_{high}$ and the inverse transformation of as shown in Equation (3):

(3)


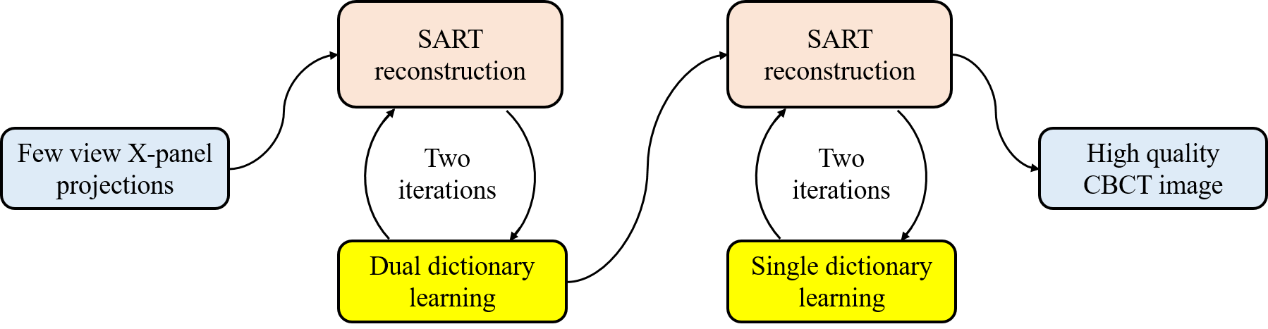


Additional figure 1 The flowchart of SART- DDL algorithm.

**The parallelization scheme of SART-DDL**

we provide the parallelization scheme for OMP. There are mainly two steps in the OMP method:

Step 1. **Adopt** the closest matching atom to the patch residue among the whole dictionary, **orthogonalize** it to all the previously adopted atoms, and **normalize** this new atom.

Step 2. Update the patch residue using the normalized atom in step 1.

Step 2 could be easily solved by an inner product of the patch residue and the normalized atom. Hence we only provide the detailed parallelization strategy for step 1.

Note the target image patch is $P$, dictionary is $D$ comprised of atoms $d_{i}$. Assume that $k$ atoms are already adopted with their representation weightings $\alpha_{i},(i=1...k)$. Thus the patch residue could be described as:

$r_{k}=P-\sum_{i=1}^{k} \alpha_{i}\cdot d_{i}$ (4)

Thus the solution of DDL is the representation weightings $\{\alpha_{i}\}$.

**The closest matching atom in Step 1**

We define the closest atom as the maximal inner product value with $r_{k}$:

$d_{k+1}=D\{\max\text{Id}(r_{k}^{T}\cdot D)\}$ (5)

where $\max\text{Id}(\cdot)$ returns the index of the maximal value of the inner product. $D\{i\}$ extracts $i\text{th}$atom in the dictionary . The calculation involves multiplying a matrix and searching the maximum. Its time complexity is $O(m\times n)$.

**Atom orthogonalization in Step 1**

Commonly the residue is updated by a Moore-Penrose generalized inverse matrix of the adopted atom group $\{d_{i}\}_{1\sim k+1}$ with newly added atom $d_{k+1}$:

$\begin{matrix} \min||r_{k}-\{d_{i}\}_{1\sim k+1}\cdot\alpha||_{2}^{2} \\ r_{k+1}=r_{k}-\{d_{i}\}_{1\sim k+1}\cdot(\{d_{i}\}_{1\sim k+1}^{T}\cdot\{d_{i}\}_{1\sim k+1})^{-1}\cdot\{d_{i}\}_{1\sim k+1}^{T}\cdot r_{k} \end{matrix}$ (6)

In order to reduce the calculations, orthogonal atoms $\{d_{i}^{new}\}_{1\sim k}$ are adopted instead of original atoms $\{d_{i}\}_{1\sim k}$ to avoid calculating Moore-Penrose generalized inverse matrix. To obtain the contribution of $d_{k+1}$ to reduce the residue $r_{k}$, we should separate the unique dimension in $d_{k+1}$ compared with the hyperspace of the span of adopted atoms, which means $d_{k+1}$ is orthogonal to . Thereafter the weight $\alpha_{k+1}$ of $(k+1)\text{th}$ atom in equation (4) can be obtained by projecting $r_{k}$ on $d_{k+1}^{new}$. The orthogonalization of $d_{k+1}$ is as follow:

$d_{k+1}=\sum_{i=1}^{k} \beta_{i}\cdot d_{i}+\Delta d,s.t.\forall i,d_{i}^{T}\cdot\Delta d=0$ (7)

where $\Delta d$ is the unique basis orthogonal to all other adopted atoms $\{d_{i}\}_{1\sim k}$ and is denoted as the new atom $d_{k+1}^{new}$ for representation. It should be noted that the representation weightings $\{\alpha_{i}\}$ are all updated when a new atom is adopted. The corresponding increment is $\{-\beta_{i}\}$. Time complexity of the orthogonalization processing is $O(k\times m)$.

**Atom normalization in Step 1**

Normalization of $d_{k}$ is as simple as following equation:

$d_{k}'=d_{k}/\sqrt{d_{k}^{T}\cdot d_{k}}$ (8)

Its time complexity is $O(m)$ and much less than the previous calculation.

Additional File Table 1 Flowchart of the algorithms for comparison.

| SART-DDL | SART-TV | FDK |
| --- | --- | --- |
| 1. Perform SART iterations to reconstruct the LD-CBCT image from few-view raw projection data. Each SART iteration includes a forward-projection of the image and a backward-projection of the deviation between raw data and the currently projected data. | 1. Perform SART iterations to reconstruct the LD-CBCT image from few-view raw projection data. | 1. Perform filtration on the few-view raw projection data per detector row and projection view. A ramp filter is adopted for the filtration. |
| 2. Perform DDL to restore the structural features in the LD-CBCT image using high-quality. Image patches in the LD-CBCT are learned by the low-quality dictionary to yield the sparse representations. Then the high-quality dictionary restore the image using the learned representations. | 2. Perform TV constraint on the LD-CBCT image. TV constraint is of the image is an objective function and could be solved by gradient descent method. The iteration step of the gradient is adaptive, and the iteration number is fixed. | 2. Weight the filtered raw data according to its projection geometry. |
| 3. Repeated step 1 and 2 for a fixed iteration number. | 3. Repeated step 1 and 2 for a fixed iteration number. | 3. Perform the backward projection of the new filtered raw data. Multiply a fixed view angle increment to correct the reconstruction values. |

Additional File Table 2 The dose parameters of the region of interest (ROI) from the original plan and the shifted plan

| The region of interest | Plan | Dose (Gy) | | | | | | |
| --- | --- | --- | --- | --- | --- | --- | --- | --- |
|  |  | D99 | D98 | D95 | Average | D50 | D2 | D1 |
| PTV7392 | Phase1 | 72.7 | 72.9 | 73.92 | 77.88 | 78.05 | 81.49 | 81.91 |
|  | Phase1shift | 72.91 | 73.16 | 73.92 | 77.89 | 78.06 | 81.42 | 81.53 |
| PTV6996-1 | Phase1 | 68.1 | 69.06 | 70.32 | 74.06 | 74.13 | 78.53 | 79.04 |
|  | Phase1shift | 67.7 | 68.85 | 70.24 | 74.04 | 74.09 | 78.54 | 78.96 |
| PTV6996-2 | Phase1 | 65.21 | 66.44 | 68.62 | 74.42 | 74.48 | 79.9 | 80.66 |
|  | Phase1shift | 66 | 67.17 | 68.96 | 74.24 | 74.43 | 79.89 | 80.51 |
| PTV5940 | Phase1 | 58.48 | 60.6 | 63.4 | 72.25 | 73.12 | 79.46 | 80.27 |
|  | Phase1shift | 57.66 | 60.09 | 63.19 | 72.18 | 73.13 | 79.43 | 80.24 |
| PTV5610-1 | Phase1 | 51.93 | 54.71 | 57.59 | 69.58 | 71.17 | 79 | 79.81 |
|  | Phase1shift | 51.81 | 54.67 | 57.68 | 69.66 | 71.31 | 78.96 | 79.79 |
| PTV5610-2 | Phase1 | 52.97 | 54.2 | 55.97 | 63.39 | 62.35 | 76.2 | 77.08 |
|  | Phase1shift | 51.89 | 53.52 | 55.51 | 63.21 | 63.21 | 76.14 | 77.04 |
| Brain Stem | Phase1 | 9.08 | 9.98 | 11.19 | 28.56 | 27.87 | 52.21 | 54.27 |
|  | Phase1shift | 10.01 | 10.68 | 11.87 | 29.74 | 28.86 | 54.12 | 56.39 |
| Brain Stem PRV | Phase1 | 8.62 | 9.66 | 11.06 | 30.33 | 29.14 | 58.52 | 60.62 |
|  | Phase1shift | 9.61 | 10.4 | 11.8 | 31.52 | 29.96 | 60.16 | 62.62 |
| The left ear | Phase1 | 38.83 | 39.47 | 42.58 | 52.58 | 50.99 | 71.12 | 72.99 |
|  | Phase1shift | 41.71 | 41.97 | 43.91 | 53.51 | 52.04 | 70.48 | 72.95 |
| The right ear | Phase1 | 26.68 | 27.23 | 27.6 | 37.21 | 35.92 | 55.06 | 56.99 |
|  | Phase1shift | 27.7 | 28.14 | 28.69 | 40.32 | 38.8 | 60.88 | 63.42 |
| The left eye | Phase1 | 0 | 0 | 3.95 | 9.98 | 6.89 | 34.6 | 36.35 |
|  | Phase1shift | 0 | 0 | 4.37 | 11.75 | 7.64 | 41.19 | 42.95 |
| The right eye | Phase1 | 3.82 | 4 | 4.32 | 9.21 | 6.57 | 28.38 | 30.62 |
|  | Phase1shift | 4.12 | 4.32 | 4.61 | 10.61 | 7.34 | 32.19 | 34.76 |
| Larynx | Phase1 | 21.6 | 22.36 | 23.67 | 35.3 | 35.3 | 56.54 | 58.86 |
|  | Phase1shift | 21.35 | 22.08 | 23.55 | 34.91 | 34.68 | 55.88 | 58.61 |
| The left len | Phase1 | 5.26 | 5.26 | 5.26 | 5.66 | 5.64 | 6.19 | 6.22 |
|  | Phase1shift | 5.45 | 5.45 | 5.46 | 5.89 | 5.86 | 6.54 | 6.57 |
| The left len PRV | Phase1 | 4.85 | 4.86 | 4.92 | 5.96 | 5.97 | 7.45 | 7.23 |
|  | Phase1shift | 5.08 | 5.13 | 5.21 | 6.28 | 6.11 | 8.28 | 8.45 |
| The right len | Phase1 | 4.68 | 4.68 | 4.68 | 5.07 | 5.02 | 5.69 | 5.83 |
|  | Phase1shift | 4.83 | 4.83 | 4.84 | 5.35 | 5.23 | 6.35 | 6.62 |
| The right len PRV | Phase1 | 4.41 | 4.44 | 4.53 | 5.44 | 5.27 | 7.95 | 8.48 |
|  | Phase1shift | 4.58 | 4.61 | 4.73 | 5.82 | 5.57 | 9.52 | 9.89 |
| The left mandible | Phase1 | 27.84 | 28.84 | 30.79 | 44.94 | 44.32 | 63.12 | 64.24 |
|  | Phase1shift | 27.63 | 28.6 | 30.58 | 44.95 | 44.1 | 63.51 | 64.69 |
| The right mandible | Phase1 | 24.96 | 26.72 | 29.29 | 37.4 | 37.91 | 52.89 | 54.89 |
|  | Phase1shift | 25.25 | 26.81 | 29.88 | 38.66 | 37.99 | 54.02 | 57.09 |
| Optic chiasma | Phase1 | 37.75 | 40.22 | 42.33 | 58.11 | 59.79 | 67.33 | 67.74 |
|  | Phase1shift | 46.96 | 50.13 | 51.74 | 61.95 | 63.88 | 67.97 | 67.97 |
| The left optic nerve | Phase1 | 17.13 | 22.54 | 30.82 | 56.49 | 61.1 | 69.07 | 69.74 |
|  | Phase1shift | 27.29 | 33.17 | 40.13 | 60.01 | 63.74 | 71.79 | 72.24 |
| The right optic nerve | Phase1 | 26.9 | 30.68 | 31.92 | 48.76 | 49.38 | 62.78 | 62.82 |
|  | Phase1shift | 29.81 | 34.59 | 36.79 | 52.23 | 52.25 | 65.12 | 66.19 |
| The left parotid | Phase1 | 17.78 | 18.21 | 19.3 | 39.19 | 33.57 | 72.26 | 74.23 |
|  | Phase1shift | 17.55 | 18.2 | 19.28 | 39.08 | 33.28 | 71.66 | 73.52 |
| The right parotid | Phase1 | 16.76 | 17.41 | 19.07 | 37.37 | 32.75 | 71.73 | 73.8 |
|  | Phase1shift | 16.78 | 17.3 | 18.98 | 38.19 | 33.35 | 73.71 | 74.88 |
| Spinal cord | Phase1 | 0 | 0 | 0 | 21.7 | 21.98 | 37.58 | 38.19 |
|  | Phase1shift | 0 | 0 | 0 | 21.71 | 22.14 | 37.69 | 37.9 |
| Spinal cord PRV | Phase1 | 0 | 0 | 0.01 | 22.97 | 23.58 | 38.03 | 38.56 |
|  | Phase1shift | 0 | 0 | 0.01 | 22.98 | 23.72 | 37.99 | 38.55 |
| The left temporal lobe | Phase1 | 2.67 | 2.98 | 3.76 | 27.18 | 21.72 | 72.94 | 74.38 |
|  | Phase1shift | 2.97 | 3.38 | 4.24 | 28.97 | 23.37 | 73.62 | 75.08 |
| The right temporal lobe | Phase1 | 2.48 | 2.67 | 3.25 | 21 | 17.3 | 59.09 | 62.81 |
|  | Phase1shift | 2.63 | 2.92 | 3.57 | 22.56 | 18.93 | 61.58 | 65.46 |
| The left temporo-mandibular joint | Phase1 | 25.41 | 25.81 | 26.3 | 39.01 | 36.02 | 65.52 | 67.92 |
|  | Phase1shift | 25.51 | 25.99 | 26.71 | 39.57 | 36.21 | 67.74 | 69.87 |
| The right temporo-mandibular joint | Phase1 | 28.24 | 28.47 | 29.38 | 38.29 | 37.7 | 53.46 | 54.5 |
|  | Phase1shift | 28.21 | 28.69 | 29.97 | 39.78 | 39.06 | 58.11 | 60.64 |
| Pituitary | Phase1 | 63.83 | 64.95 | 65.55 | 71.02 | 69.7 | 76.2 | 76.27 |
|  | Phase1shift | 68.12 | 68.97 | 69.3 | 74.5 | 73.84 | 78.87 | 78.9 |

PTV: planning target volume.

PTVn: planning target volume with the description of n cGy.

Dn: the dose that covers n% of the region of interest.

Average: average dose of the region of interest.





Additional Figure 2 The dose difference maps between the original plan and the shifted plan.
